# Antipsychotics in elderly people: to prescribe or to ban?

**DOI:** 10.1192/j.eurpsy.2023.1991

**Published:** 2023-07-19

**Authors:** M. Zbidi, W. Bouali, M. Kacem, W. Haouari, L. Zarrouk

**Affiliations:** 1 Psychiatry department, Taher sfar University hospital of mahdia tunisia, Mahdia, Tunisia

## Abstract

**Introduction:**

The prescription of psychotropic drugs is a major health problem , especially in the elderly. In fact, many studies highlight the misuse of psychotropic drugs and in particular the over-prescription of antipsychotics in the elderly which would be deleterious and not indicated.

**Objectives:**

To evaluate the prescription of antipsychotics in hospitalized elderly people in a psychiatric environment and to compare them with data from the literature.

**Methods:**

This is a retrospective descriptive study of patients aged over 65, hospitalized in the psychiatry department between January 2017 and December 2021 and who received first- or second-generation antipsychotic treatment during their hospitalization.

**Results:**

Our sample consisted of 20 patients. More than half of our sample (55%, N=11) had at least one somatic history. More than 20% of subjects, was polymedicated; and for only one patient, the ECG showed an elongation of the space QT counter indicating the use of antipsychotics. The most common diagnosis found was schizophrenia with a rate of 35%,followed by paranoia (20%), and chronic hallucinatory psychosis (15%). More than a quarter of our sample (30%, N=6) received antipsychotic treatment of first generation (AP1G), 10 patients (50%) received antipsychotic treatment of second generation(AP2G) and three patients (15%) received a combination of AP1G and AP2G. More than a quarter of our patients (30%, N=6) reported adverse effects due to neuroleptic treatment.

**Conclusions:**

The results of our study highlighted different indications for which an antipsychotic treatment was prescribed for an elderly person despite a ground often flawed, polymedicated and where the undesirable effects are superimposed.

**Disclosure of Interest:**

None Declared

